# Revealing the early stages of carbamazepine crystallization by cryoTEM and 3D electron diffraction

**DOI:** 10.1107/S2052252521010101

**Published:** 2021-10-30

**Authors:** Edward T. Broadhurst, Hongyi Xu, Simon Parsons, Fabio Nudelman

**Affiliations:** a EaStCHEM School of Chemistry and Centre for Science at Extreme Conditions, The University of Edinburgh, King’s Buildings, West Mains Road, Edinburgh EH9 3FJ, United Kingdom; b Materials and Environmental Chemistry, Stockholm University, Stockholm, SE-106 91, Sweden

**Keywords:** crystal nucleation, 3D electron diffraction, polymorphism, cryoTEM, carbamazepine

## Abstract

Time-resolved carbamazepine crystallization from wet ethanol has been monitored using a combination of cryoTEM and 3D electron diffraction. Carbamazepine is shown to crystallize exclusively as a dihydrate after 180 s. When the timescale was reduced to 30 s, three further polymorphs could be identified. At 20 s, the development of early stage carbamazepine dihydrate was observed through phase separation. This work reveals two possible crystallization pathways present in this active pharmaceutical ingredient.

## Abstract 2

A time-resolved crystallization study of early-stage carbamazepine crystallization by cryoTEM reveals two crystallization pathways, one classical and the other non-classical. Four forms of carbamazepine are also identified via 3D electron diffraction from a single crystallization.

## 1. Introduction   

Polymorphism occurs when a material crystallizes into more than one distinct solid form. It is very common in organic chemistry, affecting 74% of a set of pharmaceutical materials in one study for which extensive screening had been carried out (Cruz-Cabeza *et al.*, 2015 ▸). Polymorphs differ in solubility, bioavailability and processing (*e.g.* tableting) characteristics, and their evolution during crystal growth and storage is a complex but fundamental question worth many billions of dollars to sectors such as opto-electronics, energy storage and pharmaceuticals (Cruz-Cabeza *et al.*, 2015 ▸).

The initial stage of crystal growth, which is stochastic at an atomistic or molecular level (Gladkov, 2008 ▸), has been observed recently in the nucleation and growth of an NaCl nanocrystal at the tip of a carbon nanotube (Nakamuro *et al.*, 2021 ▸). This experiment showed an ordered nucleus emerging directly at the point of nucleation. Alternative ‘non-classical’ crystallization pathways involve initial formation of an amorphous particle which grows via the attachment of other particles (De Yoreo *et al.*, 2015 ▸).

The attaching particles may be ions and ion complexes, droplets or other amorphous or nanocrystalline materials (De Yoreo *et al.*, 2015 ▸). Work on aragonite growth, which follows this pathway, has shown that partially aligned nanocrystalline domains spontaneously and simultaneously emerge within the amorphous framework, subsequently maturing to yield a crystal (Walker *et al.*, 2017 ▸). These pathways have been extensively studied in inorganic minerals (De Yoreo *et al.*, 2015 ▸), but information on organic systems is sparse.

In this paper we describe the use of cryo-transmission electron microscopy (cryoTEM) (Dubochet *et al.*, 1988 ▸) to capture the earliest stages of crystallization of the polymorphic pharmaceutical carbamazepine. Each stage was flash-frozen in liquid ethane at 100 K after crystallization times of 20–180 s. CryoTEM allows real space imaging of crystals and the measurement of diffracted intensities in reciprocal space via tilting of the sample in the electron beam, known as three-dimensional electron diffraction (3D ED) (Wan *et al.*, 2013 ▸; Huang *et al.*, 2021 ▸; Gemmi *et al.*, 2019 ▸; Clabbers & Xu, 2020 ▸; Jones *et al.*, 2018 ▸; Gruene *et al.*, 2018 ▸). Subsequent crystal structure determinations on crystallites measuring only a few hundred nanometres lead to unambiguous polymorph identification (Broadhurst *et al.*, 2020 ▸). This combination of imaging and diffraction reveals not only short-lived polymorphs, but also the non-classical mechanism of crystal growth in this material (Walker *et al.*, 2017 ▸).

Carbamazepine [CBZ, 5H-dibenz(b,f)azepine-5-carboxamide] is a neuralgic drug which is often used as a model for polymorphism studies. There are five unsolvated polymorphs (Table S1 of the supporting information): triclinic form I (Grzesiak *et al.*, 2003 ▸), trigonal form II (Lowes *et al.*, 1987 ▸), monoclinic form III (Fernandes *et al.*, 2007 ▸) and IV (Lang *et al.*, 2002 ▸) and the most recent polymorph discovered orthorhombic form V (Arlin *et al.*, 2011 ▸). The relative stability is III > I > V > IV > II (Table S2). In the presence of water, either aqueous solution or wet solvents such as bench ethanol, a dihydrate (CBZDH) is formed (Kahela *et al.*, 1983 ▸; Kaneniwa *et al.*, 1987 ▸; Young & Suryanarayanan, 1991 ▸; Kobayashi *et al.*, 2000 ▸). CBZDH is usually described as monoclinic, though recent work has defined a disordered orthorhombic model (Sovago *et al.*, 2016 ▸). Form II also contains cavities which can accommodate solvent (Fabbiani *et al.*, 2007 ▸; Cabeza *et al.*, 2007 ▸).

## 2. Experimental   

CBZ was obtained from Alfa Aesar, with a sample purity quoted as ACS reagent grade (≥98.5%); impurities were identified using ICP-MS. Ethanol was obtained from Fisher Chemical at analytical reagent grade (≥99.8%). The characterization of water content was achieved by Karl–Fischer titration, carried out using a Mettler Toledo C30S Coulometric KF titrator equipped with a Mettler Toledo DM 143-SC electrode. Hydranal Coulomat AD was used as the solvent. Details of the ICP-MS and Karl–Fischer titrations are available in the supporting information.

In a typical experiment, a saturated solution of CBZ (0.1125 g) in ethanol (25.6257 g, water content 0.03% by Karl–Fischer titration, Table S3) was filtered under gravity to remove any undissolved CBZ. Fresh solutions were made for each study. Aliquots of 3 µl solution were pipetted onto a cryoTEM grid (Quantifoil R2/2) which had been previously plasma-treated using a PELCO Easiglow discharge cleaning system for 45 s to improve hydro­philicity. The grids were allowed to stand under ambient conditions (298 K, 21% humidity) for periods between 20 and 180 s. The ethanol was removed by pressure-assisted blotting (Zhao *et al.*, 2021 ▸) at different time-points and the sample immediately vitrified in liquid ethane to arrest further crystallization and protect the crystals from beam and vacuum damage when under the microscope. Fig. S1 of the supporting information summarizes the procedure, in which rapid blotting was accomplished using a disk of filter paper secured with a rubber band over the top of a Büchner flask connected to a water aspirator.

A Tecnai F20 FEG transmission electron microscope operating at 200 kV (λ = 0.02508 Å) equipped with a CMOS TVIPS F816 camera (8k × 8k pixels) was used for imaging and 3D ED in selected area electron diffraction (SAED) mode. A Gatan tomography cryo-transfer holder was used, operating at 100 K. During typical 3D ED data collection, diffraction patterns were collected while rotating the crystal continuously, going from −40 to +20° (Dubochet *et al.*, 1988 ▸; Clabbers & Xu, 2020 ▸; Broadhurst *et al.*, 2020 ▸; Gemmi *et al.*, 2019 ▸; Huang *et al.*, 2021 ▸). The exposure time (0.2 s) and rotation speed (0.95° s^−1^) were chosen so that individual diffraction images were integrated over 0.21° of reciprocal space. The estimated dose rate was 2 e Å^−2^ s^−1^, with each collection taking on average 240 s, the total estimated dose per 3D ED data collection was 480 e Å^−2^. The diffraction patterns were indexed and integrated with the programs *REDp* (Wan *et al.*, 2013 ▸), *XDS* (Kabsch, 2010 ▸) and *PETS* (Palatinus *et al.*, 2019 ▸). Details of structure analysis and listings of crystal and refinement data are available in the supporting information.

Samples were also prepared after 3 min and 30 s and measured using powder X-ray diffraction, further details are provided in the supporting information.

## 3. Results and discussion   

**Figure 1:**
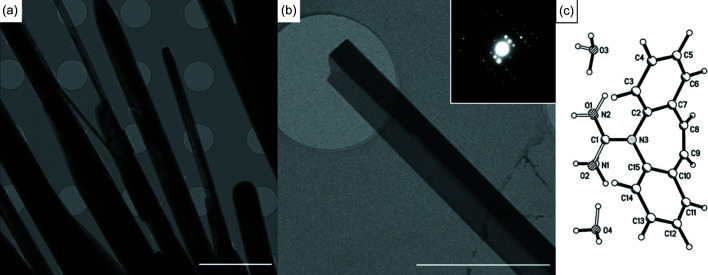
(*a*) Low-magnification image of CBZDH crystallized after 3 min on the TEM grid at 100 K showing an elongated morphology. Scale bar = 5 µm. (*b*) Selected crystal of CBZDH used for collection of 3D ED data. Scale bar = 2 µm. Inset shows a diffraction image from the dataset. (*c*) Disordered structure of CBZDH.

After 180 s, crystals with an elongated morphology [Fig. 1 ▸(*a*)] had formed across the entire grid. 3D ED data were collected from seven different crystals (Fig. S2), exhibiting typical monoclinic unit-cell dimensions of *a* = 10.38, *b* = 27.79, *c* = 5.08 Å, β = 102.52°, characteristic of the dihydrate, CBZDH. Data collected from seven crystals were merged to produce a single dataset of 90% completeness, from which the structure of CBZDH was solved and refined (*R*_1_ = 16.31%). The high value of the *R* factor reflects the neglect of dynamical effects during refinement (Clabbers & Xu, 2020 ▸) (Table S4), though the data quality was good enough for the hydrogen atoms to be located in a Fourier difference map (Fig. S3). CBZDH was the only phase observed after a crystallization time of 180 s. It has a distinctive morphology which allowed it to be identified in all other samples, both repeats and at shorter time-points.

**Figure 2:**
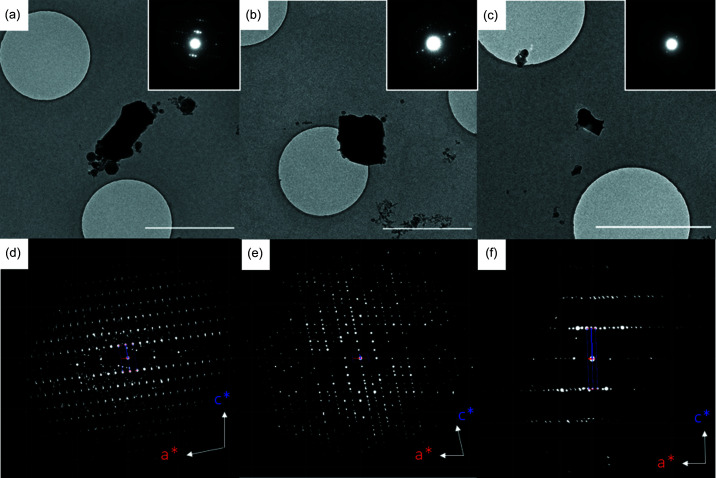
(*a*)–(*c*) Crystals of form III, IV and II, respectively, found in a sample after crystallization for 30 s on a TEM grid. Scale bars = 2 µm. The insets show representative frames from the data collections. (*d*)–(*f*) Corresponding 3D ED reciprocal lattice reconstructions all viewed along b*. All images were measured at 100 K.

To probe earlier time points of crystallization, a sample was examined at 100 K after 30 s. At this crystallization stage, no less than four different forms of CBZ were present and identified based on their unit-cell dimensions (Table S5). Alongside CBZDH, which was the dominant form, one crystal of CBZ-III with a somewhat indistinct morphology was identified from its monoclinic unit-cell dimensions *a* = 7.61, *b* = 11.30, *c* = 13.89 Å, β = 92.43° [Fig. 2 ▸(*a*)]. 3D ED data were collected to 52% completeness from this crystallite. Despite the low data completeness, the structure of CBZ-III could be solved and refined (*R*_1_ = 19.68%). Two more monoclinic crystals exhibited unit-cell dimensions of *a* = 27.15, *b* = 7.30, *c* = 14.10 Å, β = 110.30°, corresponding to CBZ-IV [Fig. 2 ▸(*b*)]. Merging of the two 3D ED datasets (66% completeness) enabled the structure to be refined (*R*_1_ = 19.86%). A third crystalline form present in the same sample exhibited rhombohedral unit-cell dimensions of *a* = 36.24, *c* = 5.31 Å, α = β = 90°, γ = 120° indicative of CBZ form II [Fig. 2 ▸(*c*)]. Although the quality of data obtained precluded structure solution, the crystal form was unambiguously identified from the unit-cell dimensions.

The identification of forms II, III and IV is the first time that any polymorph other than the dihydrate has been observed in the crystallization of CBZ from (wet) ethanol. The fact that they were only present as tiny fractions of samples otherwise consisting exclusively of the expected dihydrate demonstrates the ability of cryoTEM and 3D ED to identify minor phases and thier potential importance in polymorph discovery. The results obtained after 180 s, which showed the presence only of CBZDH, showed that these minor phases are short-lived and re-dissolve or transform into CBZDH in a matter of minutes.

To obtain bulk measurements of the crystals forming in the solution after 3 min and 30 s of crystallization, powder X-ray diffraction data were collected. At both time points, the only form detected was CBZDH (Figs. S6 and S7). The absence of the other forms at 30 s is possibly due to them being present as crystallites that are too small and too few in numbers to be detected by powder X-ray diffraction. These findings do illustrate the power of ED for phase identification within a small quantity of sample. Further details are provided in the supporting information.

**Figure 3:**
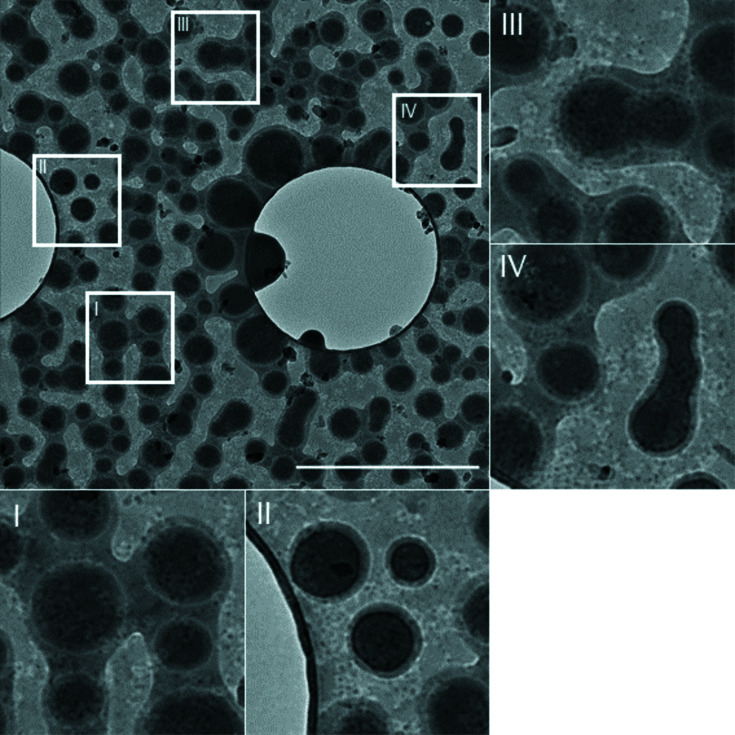
Images taken at 100 K after crystallization for 20 s and vitrification. Scale bar = 2 µm. I shows the droplet-like structures with a surrounding film of mother liquor and II shows the same structures without the surrounding film. III and IV show these structures coalescing inside and outside the film.

To investigate even earlier stages of the crystallization, samples were examined after 20 s (Fig. 3 ▸). No crystals were observed. Instead, dark droplet-like structures *ca* 150–200 nm in diameter were contained within a thinner film (area I in Fig. 3 ▸). Area II shows the droplet-like particles which lack a film of the mother liquor surrounding them and are slightly darker, suggesting that they are further developed particles of CBZ (see below). Areas III and IV show these droplet-like structures coalescing inside and outside the electron-dense film. The absence of Bragg reflections in the diffraction images of these particles showed that they were amorphous, and that the sample had been captured at a pre-crystallization stage.

The images showed that CBZDH crystallizes via a non-classical phase separation mechanism that begins with the formation of an initial film which separates into droplet-like structures (areas I and II). The coalescence of these droplet-like structures (areas III and IV) then signals the next stage of crystal growth and development. The time difference between the images in Fig. 3 ▸ and those consisting of CBZDH, along with CBZ forms II, III and IV (Fig. 2 ▸) is only 10 s, indicating the time scale of the transition from the droplets to crystals.

Pressure-assisted blotting (Zhao *et al.*, 2021 ▸) removes most of the mother liquor from the grid. As the image in Fig. 3 ▸ shows, some residue remains, but crystallization is completely halted by freezing in liquid ethane 1–2 s after blotting. A further sample was prepared after 20 s of crystallization, blotted and placed into the microscope at room temperature. The TEM vacuum (≃10^−5^ Pa) also stops crystallization by removing residual solvent, but the procedure is not as quick as plunge-freezing as it involves insertion of the sample into the microscope in a non-vitrified state, so that crystal growth can continue for a few seconds longer.

**Figure 4:**
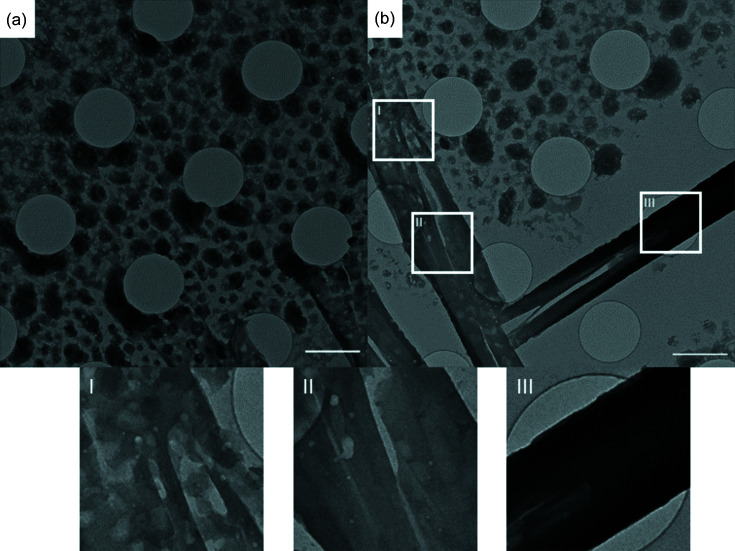
Images taken at room temperature after crystallization for 20 s and no vitrification. (*a*) Droplet-like features with roughened edges after removal of their surrounding mother liquor by the vacuum in the TEM. (*b*) Coalescence of the droplet structures producing two crystals of CBZDH, identified from morphology. White boxes labelled I, II and III show magnified areas within the sample. Scale bars = 2 µm.

Discrete, electron-dense droplet-like structures *ca* 50–150 nm in size were again present, covering the grid (Fig. 4 ▸). The existence of these features after removal of solvent demonstrates that the electron-dense droplets observed in Fig. 3 ▸ are probably amorphous CBZ particles. Although they are similar to those observed at 100 K [Fig. 3 ▸(*b*)], they now exhibit ‘roughened’ or ‘textured’ edges [Fig. 4 ▸(*a*)] as a result of solvent removal by the TEM vacuum. The images also show structures with a morphology characteristic of CBZDH [Fig. 4 ▸(*b*)], but the lack of Bragg reflections indicates that they are also non-crystalline. This absence of long-range order can be explained by dehydration of the CBZDH crystals by the high vacuum in the TEM at room temperature. Crystallinity is lost, but with preservation of morphology. The effect can be reproduced by exposing crystalline CBZDH to the TEM vacuum (Fig. S4).

Three areas of particular interest are highlighted in Fig. 4 ▸(*b*). Area I shows a part of the grid where the initial stages of the coalescence of the droplet-like particles into a pre-crystalline form of CBZ can be seen, this is comparable to what is shown in Fig. 3 ▸, areas III and IV. Fissures appearing along the length of this developing crystal are either a feature of vacuum damage as the ethanol is drawn off or that a thin crystal has been interrupted at a fragile stage of its early development. The maturing crystal is visible below this point (II), running from the left of the image down to the bottom. The more mature character of this part of the crystal is demonstrated by its more uniform texture and by the absence of particles around it, due to their incorporation into the insipient structure. The crystal in Fig. 4 ▸(*b*) area III is, by the same criteria, more mature: it is more uniform, with no fissures along its length while its darker colour indicates it is thicker. Finally, there are no roughened droplet-like structures surrounding this crystal, as found in areas I and II.

## 4. Conclusions   

Following the crystallization of CBZ using cryoTEM was made possible by combination of rapid sample preparation, imaging and 3D ED, revealing unprecedented insights into the crystal growth of an organic material. Growth begins with the formation of pre-concentrated areas of CBZ within a film of the mother liquor. Phase-separation into electron-dense, non-crystalline particles is followed by coalescence of the particles. We infer that initial crystallization occurred spontaneously in a matter of 2–3 s in some coalesced particles. Although this stage is not explicitly observed here, it has been captured in the crystallization of aragonite (Walker *et al.*, 2017 ▸).

However, the subsequent stage, consisting of a mixture of crystalline CBZDH growing by attachment of amorphous particles, has been captured by arresting growth on exposure to the TEM vacuum. Fig. 4 ▸ shows this process occurring at one end of a CBZDH crystal which is mature and fully developed at the other end.

The results establish a non-classical crystallization mechanism for CBZDH, with the whole sample becoming crystalline after only 30 s. While the bulk of the sample at 30 s consists of crystalline CBZDH, the presence of three minor forms (CBZ-II, III and IV) indicates that other crystallization routes are available. Alternative non-classical and classical growth mechanisms have been observed for CaF_2_, depending on the identity of ligands used to cap precursor nanocrystals (Mashiach *et al.*, 2021 ▸). Whether CBZ-II, III and IV develop classically or non-classically is not yet established. Here, they form as isolated crystallites (Fig. 2 ▸), lacking proximity to CBZ in other parts of the sample precluding the attachment mechanism by which CBZDH develops as discussed above. These minor forms either transform or re-dissolve, so that by 180 s the entire sample consists of crystalline CBZDH.

Finally, our results highlight the ability of time-resolved cryoTEM, coupled to 3D ED, to reveal transient phases in a crystallization process which are present in amounts too small to be detected by bulk techniques such as powder X-ray diffraction.

## 5. Related literature   

The following references are cited in the supporting information: Coelho (2018 ▸); Doyle & Turner (1968 ▸); Himes *et al.* (1981 ▸); Kachrimanis & Griesser (2012 ▸); Lisgarten *et al.* (1989 ▸); Sheldrick (2008 ▸; 2015*a* ▸); Thorn *et al.* (2012 ▸); Rigaku OD (2016 ▸).

## Supplementary Material

Crystal structure: contains datablock(s) CBZDH, CBZIII, CBZIV. DOI: 10.1107/S2052252521010101/yc5034sup1.cif

Supporting information file. DOI: 10.1107/S2052252521010101/yc5034sup2.pdf

CCDC references: 2085759, 2085760, 2085761
