# Supplementary material for: Revealing the early stages of carbamazepine crystallization by cryoTEM and 3D electron diffraction
Source: IUCrJ. 2021 Oct 30;8(Pt 6):860–6. doi: 10.1107/S2052252521010101 (PMC8562671; doi:10.1107/S2052252521010101)
Supplement: Supplementary file 2 [file m-08-00860-sup2.pdf]

IUCrJ

Volume 9 (2022)

Supporting information for article:

Revealing the early stages of carbamazepine crystallization by cryoTEM and 3D electron diffraction

Edward T. Broadhurst, Hongyi Xu, Simon Parsons and Fabio Nudelman

Experimental Procedures 2
ICP-MS 2
Notes on model refinement from electron data 3
Powder Diffraction 4

Supporting Tables 5
Table S1 5
Table S2 6
Table S3 6
Table S4 6
Table S5 7
Table S6 8

Supporting Figures 9
Figure S1 9
Figure S2 9
Figure S3 10
Figure S4 10
Figure S5 11
Figure S6 11
Figure S7 12

References 12

Experimental Procedures

ICP-MS

In addition to the development of CBZDH crystals in the samples imaged at cryogenic and RT conditions after 20 seconds of crystallization. Several very thin crystals with an unusual tear-drop morphology were also observed (Figure S5). These crystals were shown by 3DED to have a cubic *F* lattice with unit cell dimension $a = 4.21$ Å, suggestive of a simple salt. ICP-MS was carried out as described below and reveals the presence of Mg^{2+} as a trace contaminant (Table S6) and the unit cell dimensions are consistent with MgO.

Contaminants in EtOH were detected using ICP-MS using an Agilent 7500ce (with octopole reaction system), employing a radio frequency forward power of 1500 W and reflected power of 1 W, with argon gas flows of 0.8 L min^{-1} and 0.2 L min^{-1} for carrier and makeup flows, respectively. Using a peristaltic pump, sample solutions were taken up into the Mira mist nebuliser at a rate of 0.2 ml min^{-1}. Skimmer and sample cones were made of nickel. The instrument was operated in 'spectrum multi-tune acquisition mode' and three replicate runs per sample were employed. Each isotope was integrated for 0.1s per point giving a total integrations time of 0.3 s per unit mass. Three replicate runs per sample were employed to give good counting statistics. To ensure good crossover between the potential switch from pulse counting (*P*) to analogue counting (*A*) mode, a *P*/*A* factor was determined across the entire mass range at the start of each analysis day. The switch occurs at about 20-30 µg L^{-1} under the current instrument condition. The isotopes: ^{7}Li, ^{9}Be, ^{11}B. ^{23}Na, ^{24}Mg, ^{26}Mg. ^{27}Al, ^{29}Si, ^{31}P. ^{39}K, ^{42}Ca, ^{7}Li, ^{9}Be, ^{44}Ca, ^{47}Ti, ^{48}Ca, ^{49}Ti ^{51}V, ^{52}Cr, ^{53}Cr, ^{56}Fe, ^{57}Fe, ^{75}As and ^{82}Se. were analyzed in 'Helium mode' to remove any polyatomic interferences. ^{55}Mn, ^{59}Co, ^{60}Ni. ^{63}Cu, ^{66}Zn, ^{69}Ga, ^{88}Sr, ^{95}Mo, ^{107}Ag, ^{137}Ba, ^{208}Pb, ^{209}Bi and ^{238}U were analyzed in no-gas mode. ^{103}Rh was added as an internal standard spike and analyzed in both modes. In order to calibrate the instrument, a range of multi element calibration standards containing each element were prepared using two multi–element standards (Merck Multi-Element Solution VI and Merck Multi-Element Solution XVI,) plus a Rhodium single element 1000 mg L^{-1} Internal standard (SPE Science, Canada) diluted with 2% v/v HNO_3 (Aristar grade, *VWR* International, United Kingdom).

Parameters for no-gas mode:

Ion Lenses:

Extract1: 0 V

Extract 2: −133 V

Omega Bias-ce: −20 V

Omega Lens-ce: 0 V

Cell Entrance: −30 V

QP focus: 3 V

Cell Exit: −34 V

Quadrupole Parameters:

OctP Bias: −6 V

QP Bias: −3 V

Parameters for Helium Mode:

He gas flow: 6.5 ml min^{-1}

Ion lens values that differ form no-gas mode:

QP focus: 3 V

Cell Exit: −34 V

Quadrupole parameters that differ form no-gas mode:

OctP Bias: −20 V

QP Bias: −15 V

Notes on model refinement from electron data

Carbamazepine dihydrate (CBZDH) – Seven datasets were merged in XSCALE.(Kabsch, 2010) The structure was solved using SHELXT (Sheldrick, 2015a) and refined using the kinematical model of scattering in SHELXL (Sheldrick, 2015b). Molecular graphics were produced using XP (Sheldrick, 2008). The structure was modelled in space group $P2_1/c$ with the amide and solvent water disordered over two orientations. Chemically equivalent bond distances and angles were restrained to be similar in the disordered region of the structure. H atoms attached to atoms C3 – C14 were treated as variable metric rigid groups in which the CH distance was allowed to refine. Amide H-atoms in the disordered region were placed in calculated positions. H-atoms attached to water were located in a difference map. The water molecules were treated as rigid groups with hydrogen bond distances (O1 ... H3B and O2 ... H4C) restrained to the distances in the neutron structure of CBZDH (CSD refcode FEFNOT08), 1.871 and 1.910 Å, respectively. All full-occupancy atoms were refined with anisotropic displacement parameters subject to enhanced rigid-body restraints (Thorn *et al.*, 2012). Disordered atoms were refined isotropically. The final value of $R1$ was 16.31%.

Form III – A single dataset was indexed and integrated in PETS 2.0 (Palatinus *et al.*, 2019). As this was the only crystal observed, merging of data sets was not possible. Solution and refinement procedures were similar to those described above for CBZDH. H atom distances were constrained to typical electron CH and NH distances from the structure determination of glycine from electron diffraction data (refcode KUFDOH, 1.168 and 1.184 Å, respectively). CH distances involving H7, H10 and H11 were allowed to refine freely in variable metric rigid groups. The final value of $R1$ was 19.68%.

Form IV – Two datasets were merged and processed in the same way as CBZDH. Starting atomic coordinates were taken from (Lang *et al.*, 2002) (refcode CBMZPN12). Refinement procedures were similar to those described above. Most H atoms were allowed to refine freely as variable metric rigid

groups. CH distances involving H6, H8, H9, H11 and H12 were constrained to typical electron CH distance of 1.168 Å. The final value of $R1$ was 19.86%.

Electron scattering factors were taken from Doyle and Turner (Doyle & Turner, 1968). A listing of crystal and refinement statistics available in Table S4. The crystal structure information files for CBZDH, CBZIII and CBZIV are deposited with The Cambridge Crystallographic Data Centre (CCDC). (CCDC 2085759-2085761)

Powder Diffraction

Powder diffraction data was collected on two samples. For the first, a sample was prepared by allowing a 3 ml drop of the saturated carbamazepine-ethanol solution (see Section 2 of the main text) to crystallize for three minutes directly on the powder sample holder. The samples were exposed to Cu Kα radiation (30 kV, 10 mA) in a wide-angle powder x-ray diffractometer (D2 Phaser, Bruker, USA). The instrument was operated in Bragg-Brentano geometry with steps in increments of 0.08° 2θ. The angular range was 5 – 50° in 2θ and the counts were accumulated for 1 second at each step. The total time for each data collection was ten minutes.

A second sample was prepared by dipping a MiTeGen mounting loop into the saturated carbamazepine-ethanol solution and then allowing to crystallize for 30 seconds before starting the data collection on a single crystal X-ray diffractometer (Rigaku Supernova, Oxford Diffraction, UK). The instrument was operated in powder measurement mode using CrysalisPro in the default settings (CrysAlis, 2009). The total time for this data collection was 15 minutes. The sample obtained in this way consisted of a small collection of crystallites and was not large enough to study on the powder diffractometer.

Carbamazepine dihydrate (CBZDH) has a lath-like habit where the dominant face is 020 (Kachrimanis & Griesser, 2012). The powder pattern obtained after 3 min could mostly be indexed and CBZDH with 0*k*0 reflections with even values of k from 4 to 14. The pattern could be successfully fitted using the Pawley method (Coelho, 2018) assuming space group $P2_1/c$ using these together with the 140, 220, 240 and 271 reflections (Figure S6). It was not possible to refine all four cell dimensions as information in the a^*c^* plane is limited, but a = 10.155(2) Å [lit. 10.1621 Å in CSD refcode FEFNOT04], b = 28.7043(9) Å [28.705 Å] and c = 4.9181(14) Å [4.9348 Å]. The β angle was fixed to the literature value of 103.33°; refining it yielded 103.29(87.8)°. A TCHZ function was used for the peak-shape and a six-term Chebychev polynomial for the background. The R_{wp}-factor was 5.53%.

The data obtained after 30 sec could also be modelled as CBZDH. The first peaks were observed at 2θ ~20°. Several peaks below this are missing indicating that texture effects are significant. A Pawley fit (Figure S7) using cell dimensions fixed to their literature values (CSD refcode FEFNOT04) converged to R_{wp} = 1.07%. A TCHZ function was used for the peak-shape and a six-term Chebychev polynomial for the background. The profiles in the pattern are quite broad and the structures relatively complex and reasonable Pawley fits could also be obtained with other phases. Notably a fit

to CBZ-I which is even more complex than CBZDH, being triclinic with $Z = 8$, converged to R_{wp} = 1.10%. While characterisation of the 30 sec sample by this method can thus not be regarded as definitive, these findings do illustrate the power of electron diffraction for phase identification of small samples.

Supporting Tables

Table S1 Crystallographic information on the reported different polymorphs of carbamazepine and carbamazepine dihydrate (CBZDH).

Polymorph	III	II*	I	IV	V	CBZDH
Space group	$P2_1/n$	*R-3*	*P-1*	*C2/c*	*Pbca*	$P2_1/c$
a/Å	7.537(1)	35.454(3)	5.1705(6)	26.609(4)	9.1245(5)	10.144(4)
b/Å	11.156(2)	35.454(3)	20.574(2)	6.9269(10)	10.4518(5)	28.891(11)
c/Å	13.912(3)	5.253(1)	22.245(2)	13.957(2)	24.8224(11)	4.847(2)
α/°	90	90	84.124(4)	90	90	90
β/°	92.86(2)	90	88.008(4)	109.702(2)	90	103.763(11)
γ/°	90	120	85.187(4)	90	90	90
V/Å^3	1168.3	5718.32	2344.82	2421.93	2367.25	1379.73
Z	4	18	8	8	8	4
Z'	1	1	4	1	1	1
Temperature (K)	298	298	158	158	123	100
Pressure (GPa)	0	0	0	0	0	0
REFCODE	CBMZPN10	CBMZPN03	CBMZPN11	CBMZPN12	CBMZPN16	FEFNOT09
Reference	(Himes et al., 1981)	(Lowes et al., 1987)	(Grzesiak et al., 2003)	(Lang et al., 2002)	(Arlin *et al.*, 2011)	(Sovago et al., 2016)

Table S2 Lattice energy (E_{Latt}) and Internal energy (U_{inter}) of the five different polymorphs of carbamazepine (Lisgarten *et al.*, 1989).

Polymorph	E_{Latt} / kJ mol^{-1}	U_{inter} / kJ mol^{-1}	Density / g cm^{-3}
III	−128.760	−130.270	1.346
II	−121.970	−125.340	1.244
I	−124.800	−127.440	1.310
IV	−123.250	−124.980	1.259
V	−124.690	−125.500	1.296

Table S3 Results from three runs of Karl-Fischer titrations to determine the water content within the ethanol.

run	Mass of syringe + ethanol (g)	mass of syringe (g)	mass of ethanol (g)	water content (mg/L)
1	3.464	2.998	0.466	212
2	3.457	2.874	0.583	209
3	3.471	2.865	0.606	236

Mass of 1 L of wet ethanol = 789.45 g (assuming the density is the same as pure ethanol (0.78945 g cm^{-3}))
Mass of water in 1 L = 219 x 10^{-3} g (average value from runs 1 – 3)
% water = 100 x (219 x 10^{-3}/789.45) = 0.028 % water by mass.

Table S4 Experimental crystallographic and refinement data. Experiments were carried out at 100 K with electron radiation, λ = 0.02508 Å.

Polymorph	CBZDH	CBZIII	CBZIV
Crystal System	Monoclinic	Monoclinic	Monoclinic
Space group	*P2₁/c*	*P2₁/n*	*C2/c*
a, b, c (Å)	10.410 (2), 28.117 (6), 5.038 (1)	7.614 (2), 11.302 (2), 13.886 (3)	27.150 (5), 7.3000 (15), 14.100 (3)
β (°)	104.64 (3)	92.43 (3)	110.30 (3)
V (Å^3)	1426.8	1193.9	2621.0
Z	4	4	8
Z'	1	1	1

Crystal size (μm)	6 x 0.5 x 0.001	1.5 x 1.0 x 0.5	1.5 x 0.8 x 0.03
**Data collection**			
No. of measured, independent and strong reflections [*Fo* > 4σ(*Fo*)]	16902, 2602, 1576	2523, 1362, 754	4267, 1645, 738
R_{int}	0.531	0.258	0.230
Resolution (Å)	0.63	0.70	0.62
Completeness (%)	90	51	65
**Refinement**			
R1 (for all reflections), *wR2*, *S*	0.163, 0.433, 1.10	0.197, 0.542, 1.63	0.199, 0.481, 1.21
No. of reflections	2602	1362	1645
No. of parameters	196	162	171
No. of restraints	181	111	144
Final difference map extremes (e $Å^{-1}$)	0.128, −0.129	0.135, −0.148	0.142, −0.174

Table S5 3D ED Unit cell parameters determined by REDp, collected from crystals found after 30 seconds of crystallization.

sample	*a*(Å)	*b*(Å)	*c*(Å)	α(°)	β(°)	γ(°)	*V*($Å^3$)	comments
1	35.98	34.87	15.44	119.83	119.84	103.86	11117.1	A, B
2	18.38	8.02	15.59	93.08	132.6	98.71	1632.9	A
3	25.9	24.53	23.69	70.19	114.11	114.53	12238.5	A
4	73.02	67.61	23.73	66.66	113.32	157.44	41108	A
5	34.82	19.09	29.49	78.91	133.07	111.59	13269.8	A, B
6	7.41	4.70	4.56	119.74	89.81	91.23	138	ice
7	18.94	4.94	7.40	84.65	95.63	115.85	622.4	A
8	46.36	14.41	45.94	94.69	142.63	96.05	17811.6	A
9	14.08	7.52	14.09	105	108.94	91.58	1353	form IV
10	51.87	49.18	45.06	152.64	29.35	151.28	23034.6	A

11	13.89	11.35	7.66	90.82	92.47	90.04	1205.7	form III
12	20.66	20.66	5.31	94.36	94.79	117.38	2011.6	form II
13	27.17	4.84	10.22	103.7	93.26	80.58	1287.9	CBZDH
14	29	53.92	54.05	159.33	90.39	89.98	29830	A
15	14.11	7.21	14.06	103.83	109.8	90.13	1300.6	form IV
16	23.12	7.65	4.852	95.05	82.73	95.88	844.4	B
17	31.84	37.38	35.77	137.84	62.95	122.11	23721.1	A, B

A = Presence of multiple lattices within the 3D reconstruction (the crystal was a twin or conglomerate).

B = Not enough discrete reflections for reliable indexing.

Table S6 Selected ions from the ICP-MS measurements.

Isotope	^{24}Mg	^{26}Mg	^{11}B	^{23}Na	^{39}K
ICP-MS sample (μgL^{-1})	28.06	308.2	299.3	154.3	146.1
ICP-MS blank (μgL^{-1})	7.41	49.71	67.26	−76.88	−15.24
Conc of ion (μgL^{-1})	20.65	258.49	232.04	231.18	161.34
Conc of ion (gL^{-1})	0.00002065	0.000258	0.000232	0.000231	0.000161
one 3uL drop	0.000003	0.000003	0.000003	0.000003	0.000003
mass of ion in 3uL drop (g)	6.195E−11	7.75E−10	6.96E−10	6.94E−10	4.84E−10

Supporting Figures

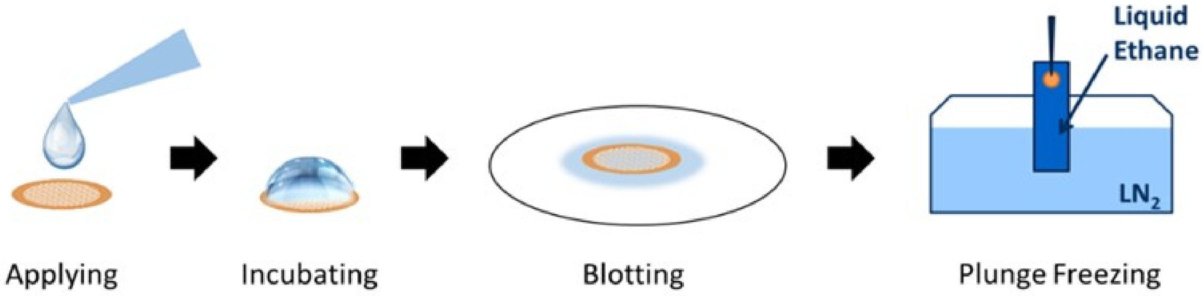

Figure S1 Schematic of sample preparation procedure used in this work.

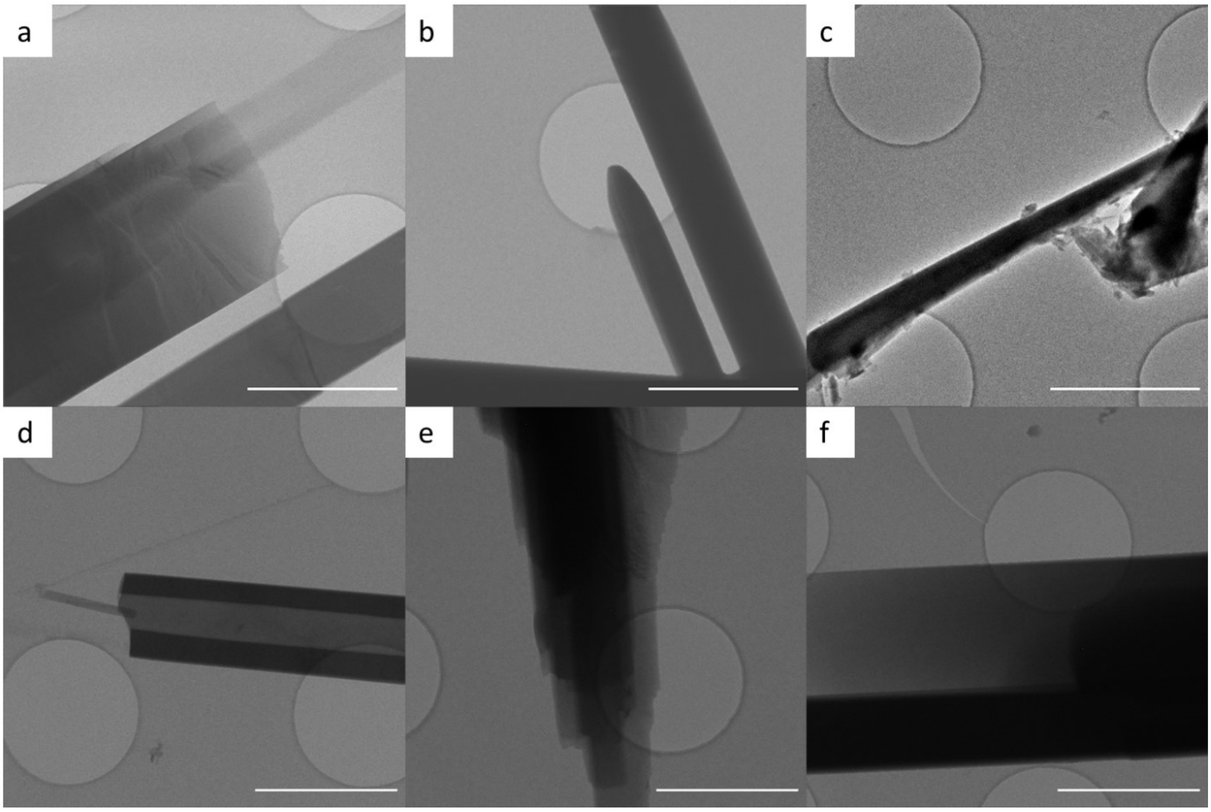

Figure S2 a – f): Images of six other crystals used for collection of diffraction data after crystallization for 180 s. CBZDH shows a distinct elongated morphology allowing easy identification from the TEM grid. All scale bars = 2 µm.

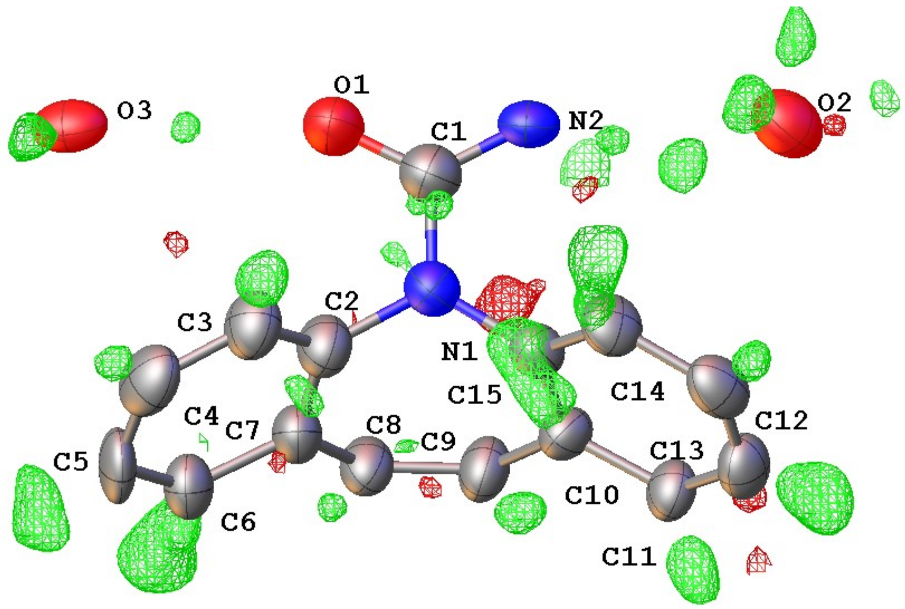

Figure S3 Fourier difference map of CBZDH showing scattering density corresponding to the hydrogen atoms (green). Contour level drawn at 0.6 e Å^3 (green).

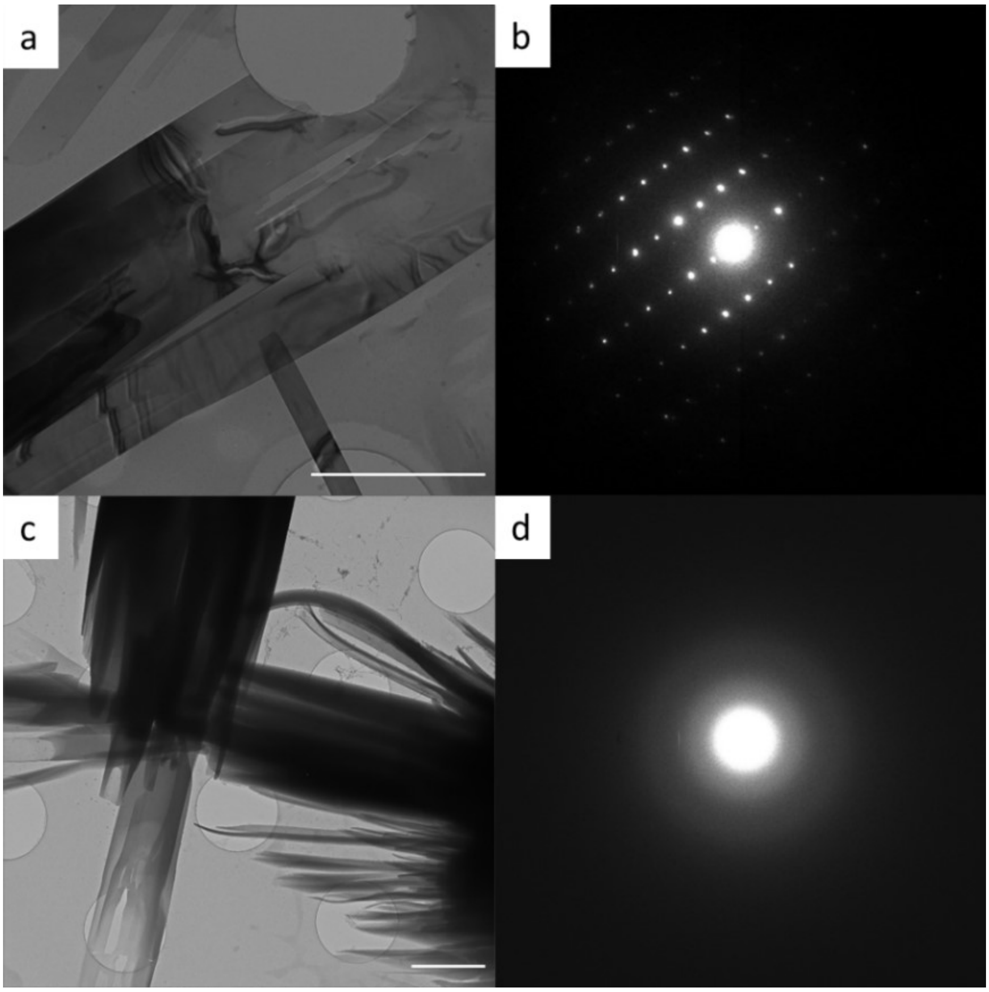

Figure S4 a) Crystal of CBZDH formed after 20 s and imaged at 100 K. b) Diffraction images were obtained with the stage at 273 K. c) After warming to 298 K, the same crystals were no longer crystalline (d), but morphology was preserved. Scale bar is 2 µm in both images.

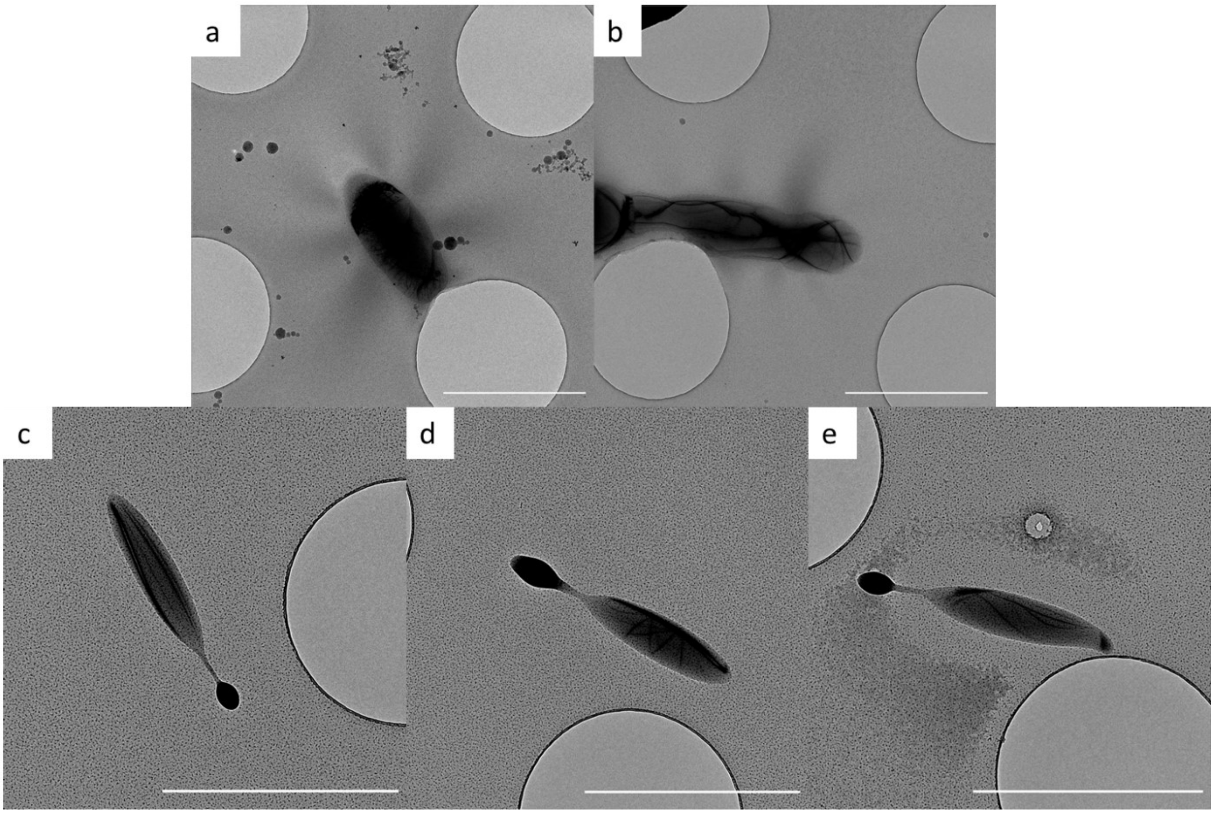

Figure S5 a – e show crystals of suspected MgO based on 3D ED and ICP measurements. These crystals were shown by 3D ED to have a cubic *F* lattice with unit cell dimension *a* = 4.21 Å, suggestive of a simple salt. ICP experiments on the sample of CBZ reveals the presence of Mg^{2+} as a trace contaminant (Table S6) and the unit cell dimensions are consistent with MgO. All scale bars = 2 µm.

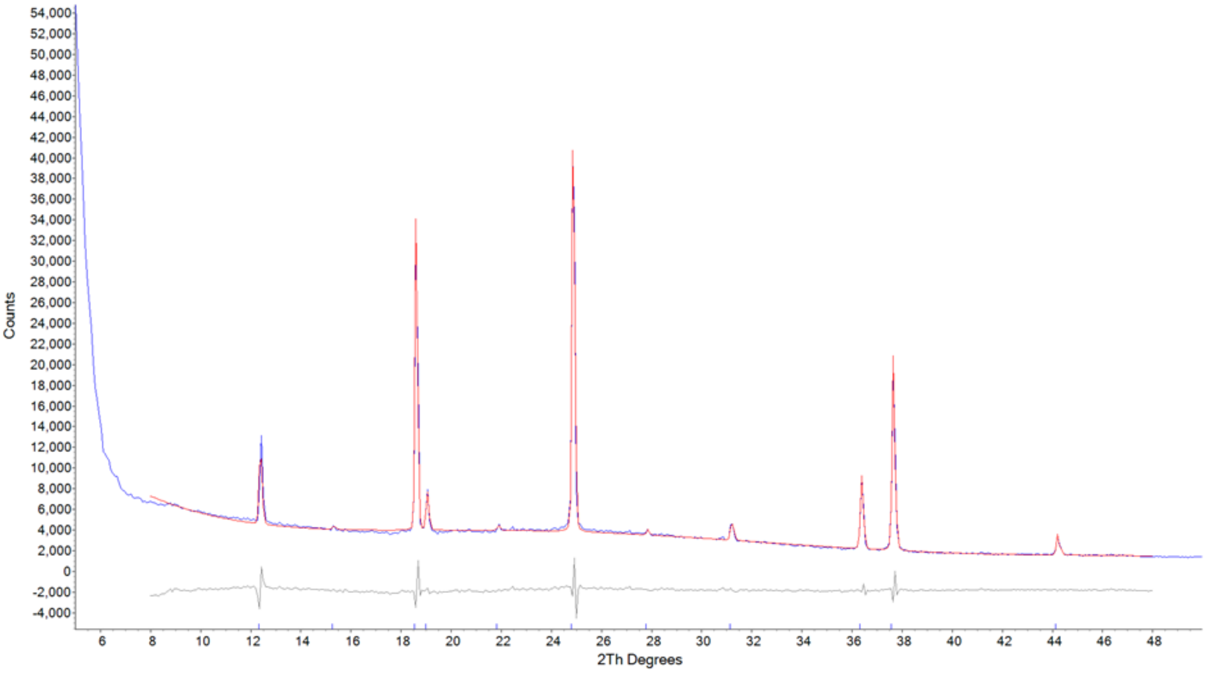

Figure S6 Pawley fit of data obtained after 3 min crystallisation using parameters for CBZDH.

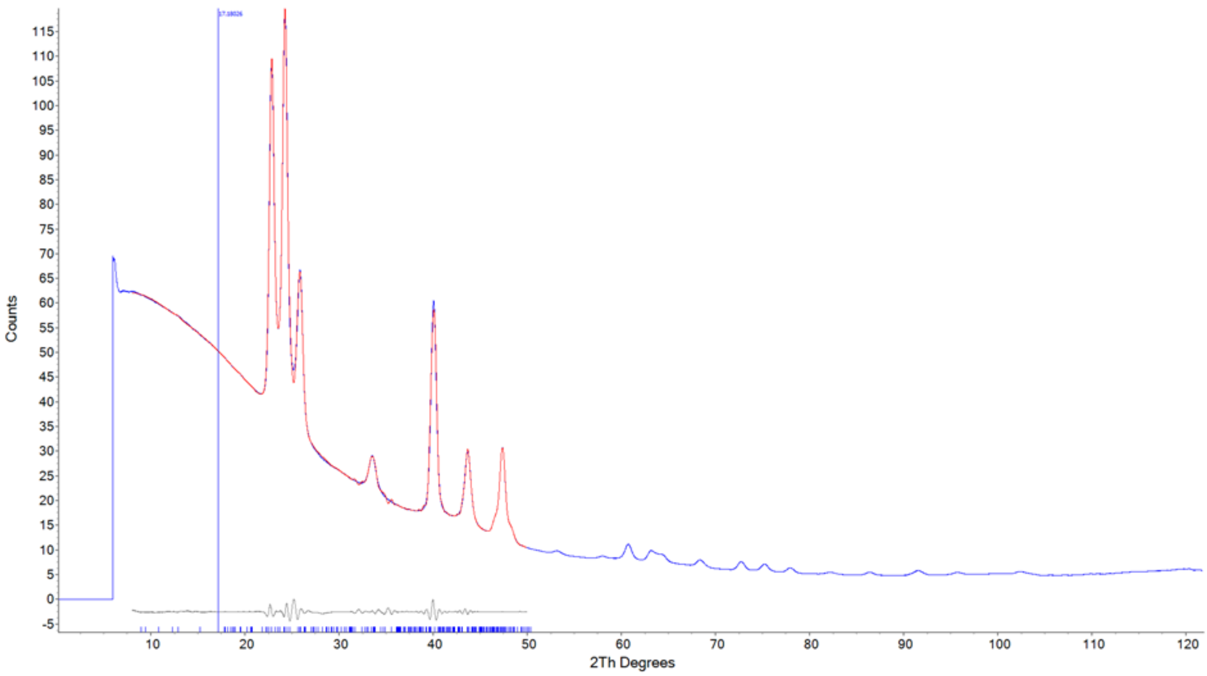

Figure S7 Pawley fit of data obtained after 30 s crystallisation using parameters for CBZDH.

References

Arlin, J. B., Price, L. S., Price, S. L. & Florence, A. J. (2011). *Chemical Communications* **47**, 7074-7076.

Coelho, A. A. (2018). Journal of Applied Crystallography **51**, 210-218.

CrysAlis, P. R. O. (2009). Yarnton, England.

Doyle, P. A. & Turner, P. S. (1968). Acta Crystallographica Section A: Crystal Physics, Diffraction, Theoretical and General Crystallography **24**, 390-397.

Grzesiak, A. L., Lang, M., Kim, K. & Matzger, A. J. (2003). Journal of Pharmaceutical Sciences **92**, 2260-2271.

Himes, V. L., Mighell, A. D. & De Camp, W. H. (1981). Acta Crystallographica Section B: Structural Crystallography and Crystal Chemistry **37**, 2242-2245.

Kabsch, W. (2010). Acta Crystallographica Section D: Biological Crystallography **66**, 125-132.

Kachrimanis, K. & Griesser, U. J. (2012). Pharmaceutical research **29**, 1143-1157.

Lang, M., Kampf, J. W. & Matzger, A. J. (2002). Journal of Pharmaceutical Sciences **91**, 1186-1190.

Lisgarten, J. N., Palmer, R. A. & Saldanha, J. W. (1989). Journal of Crystallographic and Spectroscopic Research **19**, 641-649.

Lowes, M. M. J., Caira, M. R., Lötter, A. P. & Van Der Watt, J. G. (1987). Journal of Pharmaceutical Sciences **76**, 744-752.

Palatinus, L., Brázda, P., Jelínek, M., Hrdá, J., Steciuk, G. & Klementová, M. (2019). Acta Crystallographica Section B: Structural Science, Crystal Engineering and Materials **75**, 512-522.

Sheldrick, G. M. (2008). Acta Crystallographica Section A: Foundations of Crystallography **64**, 112-122.

Sheldrick, G. M. (2015a). Acta Crystallographica Section A: Foundations and Advances **71**, 3-8.

Sheldrick, G. M. (2015b). Acta Crystallographica Section C: Structural Chemistry **71**, 3-8.

Sovago, I., Gutmann, M. J., Senn, H. M., Thomas, L. H., Wilson, C. C. & Farrugia, L. J. (2016). Acta Crystallographica Section B: Structural Science, Crystal Engineering and Materials **72**, 39-50.

Thorn, A., Dittrich, B. & Sheldrick, G. M. (2012). Acta Crystallographica Section A: Foundations of Crystallography **68**, 448-451.
